# ST-Segment-Elevation Acute Coronary Syndrome in a Patient With a Superdominant Right Coronary Artery and a Coincident Absent Left Circumflex Artery

**DOI:** 10.7759/cureus.90057

**Published:** 2025-08-14

**Authors:** Priya Ramcharan, Arun R Katwaroo, Matthew A Maharaj, Nicholas Pereira, Valmiki K Seecheran, Nafeesah Ali, Rajeev V Seecheran, Lakshmipathi Peram, Naveen A Seecheran

**Affiliations:** 1 Cardiology, Eric Williams Medical Sciences Complex, Mt. Hope, TTO; 2 Internal Medicine, St. James Medical Complex, Port of Spain, TTO; 3 Accident and Emergency, Eric Williams Medical Sciences Complex, Mt. Hope, TTO; 4 Internal Medicine, Eric Williams Medical Sciences Complex, Mt. Hope, TTO; 5 Nephrology, University of New Mexico Medical Center, Alburquerque, USA

**Keywords:** absent left circumflex (lcx) artery, acute coronary syndrome (acs), coronary artery anomaly (caa), st-segment-elevation acute coronary syndrome (ste-acs), superdominant right coronary artery (rca)

## Abstract

We describe a case of a 42-year-old Caribbean South Asian male with no conventional cardiovascular risk factors who presented with an inferolateral ST-segment-elevation acute coronary syndrome (STE-ACS) with thrombolysis in myocardial infarction (TIMI) thrombus grade 4 in a superdominant right coronary artery (sdRCA) and absent left circumflex (LCx) artery. The healthcare team should be cognizant of rare coronary artery anomalies (CAAs), their pathophysiology, and the individualized management strategies for these related ACS.

## Introduction

Acute coronary syndromes (ACS) pose a significant medical challenge, requiring emergent and optimal management to attenuate myocardial injury and improve patient outcomes [1,2]. The pathophysiology of ACS typically involves atherosclerotic plaque rupture and subsequent thrombosis [1,3,4]. Coronary artery anomalies (CAAs) are relatively rare but may prove clinically significant as their potential to influence the presentation and management of ACS necessitates heightened awareness [5,6].

CAAs encompass a diverse spectrum of congenital malformations affecting the coronary arteries’ (CAs) origin, course, and termination [5,6]. These anomalies can involve any significant coronary vessels with variable clinical significance. The rarity of CAAs often leads to a diagnostic dilemma, particularly when they present atypically or mimic the clinical presentation of more common ischemic etiologies such as ACS [7]. A superdominant right coronary artery (sdRCA) refers to an exceedingly rare variant where the RCA is exceptionally large and perfuses not only its usual territory but also a vast portion of myocardium typically supplied by the left circumflex artery (LCx). This occurs when the LCx is either absent or hypoplastic (underdeveloped) (0.003%), and the RCA compensates by providing the blood supply to the area normally supplied by the LCx [5,6]. This must be ascertained early to avoid deleterious iatrogenic injury when selecting the interventional toolkit and armamentarium, such as coronary dissection or perforation.

This case report details a young Caribbean South Asian male presenting with an acute inferolateral ST-segment elevation acute coronary syndrome (STE-ACS) despite lacking traditional coronary artery disease (CAD) risk factors, with a high thrombus burden in a sdRCA and absent LCx. This atypical presentation highlights the potential for underlying anatomical variations to influence the clinical course of ACS, a time-sensitive condition where minimizing the “door-to-balloon” time is crucial for optimizing patient outcomes and prognosis [8].

## Case presentation

A 42-year-old Caribbean South Asian male with a medical history significant for suspected anaphylaxis to aspirin and NSAIDs presented to the emergency room, reporting typical, substernal angina, which worsened with exertion and alleviated with rest for the previous three hours. It was associated with presyncope, dyspnea, and emesis; however, there were no palpitations or syncope.

**Figure 1:**
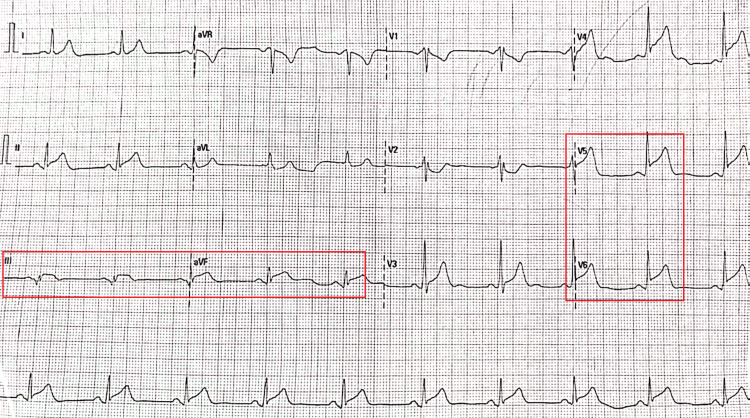
The patient’s 12-lead electrocardiogram (ECG) with the left red panel illustrating the 3-millimeter ST-segment elevation (STE) in leads III and aVF and the right red panel illustrating the 5-millimeter STE in V5 and V6, suggesting an inferolateral ST-segment acute coronary syndrome (STE-ACS).

Upon presentation, his vital signs included a blood pressure of 142/96 millimeters of mercury (mmHg), a heart rate of 88 beats per minute, a regular respiratory rate of 22 breaths per minute, with an oxygen saturation of 98% on 2 liters of oxygen via nasal cannula, and a temperature of 37 °C, which was afebrile. The patient was alert and coherent, with no focal neurological deficits. The cardiovascular examination revealed normal heart sounds (S1 and S2) without any additional sounds or murmurs. His jugular venous pulse was not elevated, and there was no sacral or pedal edema. His apical impulse was not displaced and had a normal character. Respiratory examination indicated bilateral vesicular breath sounds without wheezing or crackles. An initial 12-lead electrocardiogram (ECG) performed revealed 3-millimeter (mm) STE in leads III and aVF and 5-mm STE in V5 and V6, suggesting an inferolateral STE-ACS (Figure 1). An emergent high-sensitivity troponin I (hs-cTnI) level was significantly elevated at 4,192 ng/L (normal range: 0-20 ng/L).

**Figure 2:**
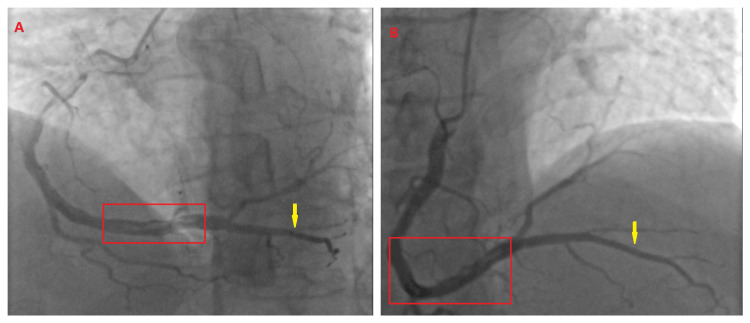
The patient’s cineangiography of the right coronary artery (RCA). (A) A left anterior oblique (LAO) view of the RCA, with the red panel illustrating the thrombolysis in myocardial infarction (TIMI) thrombus grade 4 and the yellow arrow indicating the superdominant right coronary artery (sdRCA) and posterior descending artery (PDA) system perfusing the lateral wall. (B) A right anterior oblique (RAO) view of the sdRCA, with the red panel illustrating the TIMI thrombus grade 4 and the yellow arrow indicating the sdRCA and PDA system perfusing the lateral wall.

He immediately proceeded to primary percutaneous coronary intervention (PPCI), where distal transradial coronary cineangiography (Figure 2) revealed thrombolysis in myocardial infarction (TIMI) grade 4 thrombus within the distal sdRCA of approximately 3.5 - 4.0 mm caliber and the posterior descending artery (PDA) system perfusing the lateral wall [9,10].

**Figure 3:**
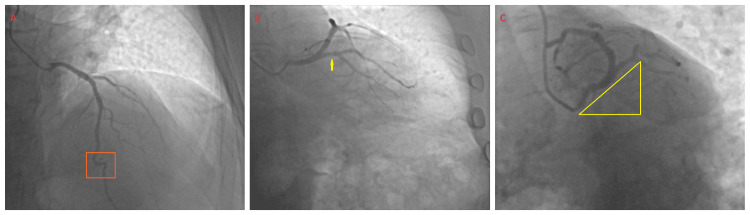
The patient’s cineangiography of the left coronary artery (LCA). (A) A straight cranial view of the patient’s left anterior descending (LAD) artery with the orange box indicating mild mid-LAD segment intramyocardial bridging without “milking” phenomenon. (B) A right anterior oblique caudal (RAO-caudal) view with the yellow arrow demonstrating the ramus intermedius (RI) artery and lack of an angiographically definitive left circumflex (LCx) artery. (C) A left anterior oblique caudal (LAO-caudal) view with the yellow triangle demonstrating the lack of an angiographically definitive LCx artery perfusing the lateral wall.

There was mild mid-left anterior descending artery (LAD) segment intramyocardial bridging without the “milking” phenomenon (Figure 3). Notably, a ramus intermedius (RI) artery was present; however, there was a lack of an angiographically definitive LCx artery. Due to the high thrombus burden, a real-time decision was made to forego aspiration thrombectomy and culprit vessel intervention, despite the TIMI 2 antegrade slow flow, in favor of pharmacological management. A low-dose intracoronary cocktail formulated by Seecheran, (Naveen, corresponding author) was catheter-infused, comprising nitroglycerin, adenosine, nicardipine, and tirofiban, in an attempt to improve flow hemodynamics transiently [11].

Post-procedure, he was admitted to the cardiac care unit (CCU), where he was initiated on comprehensive, guideline-directed medical therapy (CGDMT) for his inferolateral STE-ACS. His cardiovascular regimen included ticagrelor 90 mg twice daily, apixaban 5 mg twice daily, rosuvastatin 40 mg, ramipril 5 mg twice daily, carvedilol 6.25 mg twice daily, eplerenone 25 mg, empagliflozin 10 mg, and colchicine 0.5 mg. A bedside 2-dimensional transthoracic echocardiogram demonstrated subtle regional wall motion abnormalities consistent with RCA and LCx artery territory ischemia with a preserved left ventricular ejection fraction (LVEF ~50%), mildly reduced tricuspid annular plane systolic excursion (TAPSE ~12 millimeters), no valvular dysfunction, left ventricular mural thrombus, and pulmonary hypertension.

**Figure 4:**
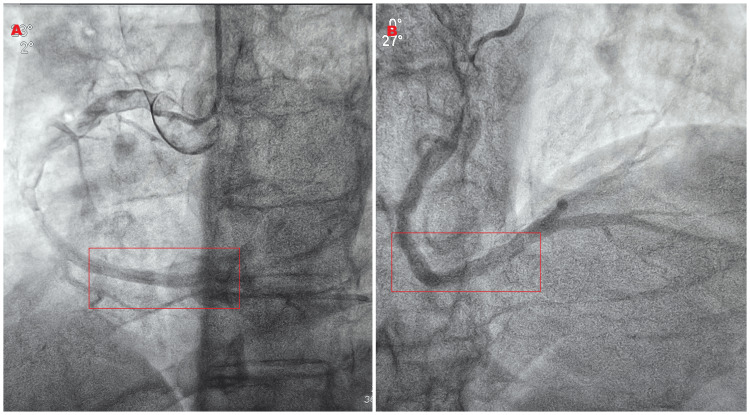
The patient's repeat coronary angiography of his superdominant right coronary artery (sdRCA) 1-month post-hospitalization. (A) A left anterior oblique (LAO)-cranial image demonstrating complete thrombus dissolution within the red rectangle. (B) A right anterior oblique (RAO)-cranial also indicating thrombus resolution within the red box.

During his ensuing hospitalization, the patient remained hemodynamically stable without any interval adverse events. His hs-cTnI levels significantly declined over the subsequent days, with readings of 28.2 ng/L on day 2 and 8.6 ng/L on day 3. His low-density lipoprotein was 164 mg/dL (normal < 130 mg/dL) in the setting of his index ACS. He was eventually discharged with a scheduled follow-up Cardiology outpatient appointment after extensive lifestyle counseling. The patient returned for repeat coronary angiography one month later, which demonstrated complete thrombus dissolution with TIMI 3 antegrade flow (Figure 4).

## Discussion

CAAs, though rare, are typically identified incidentally during coronary angiography, with a prevalence rate of approximately 1 - 1.3% [12,13]. Despite their rarity, these anomalies pose significant challenges in managing ACS, particularly when minimizing door-to-balloon time is crucial for patient care. CAs typically develop within the epicardial interventricular and atrioventricular grooves, with their proximal ends within the aortic sinuses. The RCA arises from the right sinus, while the LCA arises from the left sinus. After exiting the right sinus, the RCA traverses the right atrioventricular sulcus to the right margin and base of the heart. Its branches include the conus artery, the sinus node artery, the right marginal branch, the atrioventricular nodal branch, and the posterior interventricular branch. The left main coronary artery (LMCA) originates from the left aortic sinus and branches to give the LCx artery and the LAD artery. The LCx artery runs along the left side of the heart in the coronary sulcus, giving rise to the left marginal artery, the posterolateral branch, and the obtuse marginal (OM) branches [14]. An absent LCx artery is extremely rare, with an incidence of 0.003% [15]. In such cases, the LMCA continues as the LAD artery, with a complete absence of the LCx artery and OM branches, and the RCA becomes superdominant, with its distal branches coursing retrogradely in the left atrioventricular groove to supply the left ventricle [16]. There are only a few reported cases of absent LCx artery, with the variant usually benign and asymptomatic [17,18]. They are often discovered incidentally, such as during investigations for angina, arrhythmias, and valve surgery [19,20].

An absent LCx artery characterized by angina is usually triggered by focal ischemia in a hypoperfused zone and typically presents as an inferior or inferolateral ACS [21]. This can be attributed to the steal phenomenon with sequelae of increased metabolic demands [22]. This CAA can obfuscate management as it can be misconstrued as a total occlusion. This can angiographically appear as a 'stump,' which is a slight contrast 'hang-up' at the ostial segment [22]. This must be ascertained early to avoid deleterious iatrogenic injury when selecting the interventional toolkit and armamentarium, such as coronary dissection or perforation [22]. The absence of the LCx artery can be compensated by an oversized, sdRCA that perfuses both territories; however, this carries the potential risk of rupture, dissection, thrombosis, and embolization [23-25]. Giant CAs, such as those seen in CA ectasia, have an increased propensity for ACS, regardless of whether the CA is occluded [26-28]. This is primarily attributed to impaired hemodynamic flow and resultant thrombotic milieu in the dilated vessel [23,25,29].

One case reported the successful treatment of acute occlusion of an sdRCA using a mechanical aspiration, while another described resolution with bare-metal stents [23]. For percutaneous coronary interventions, intravascular ultrasound or optical coherence tomography imaging is recommended to deploy drug-eluting stents or perform aspiration thrombectomy optimally. However, in our local setting, these advanced intracoronary imaging modalities are not available, and thus, we could not definitively ascertain the absence of the LCx artery; instead, we relied solely on visual interpretation of the angiographic images. There is a rarity of cases primarily instituting anticoagulation as a conservative, non-invasive strategy. As our patient was aspirin hypersensitive, we opted for an advanced antithrombotic regimen that incorporated ticagrelor, a potent antiplatelet agent, and apixaban, a direct oral anticoagulant. This was initially predicated on the "What is the Optimal antiplatElet and anticoagulant therapy in patients with oral anticoagulation and coronary StenTing (WOEST)" trial [30,31]. We also adopted a modified regimen from the "The AUGUSTUS (Antithrombotic Therapy After Acute Coronary Syndrome or PCI in Atrial Fibrillation)" study, which comprised apixaban 5 mg combined with ticagrelor, which suggested a lower signal for severe bleeding episodes [32,33]. Recent international guidelines recommend that the duration of "triple therapy" be shortened and, in some instances, omitted in favor of dual antithrombotic therapy with a direct oral anticoagulant and an antiplatelet agent [32,33]. Ticagrelor and apixaban without ASA inhibit platelet activation and thrombin formation in vivo in healthy subjects [32]. We considered this a prudent strategy in the setting of possible anaphylaxis, given the short timeframe for an aspirin desensitization protocol and PPCI, and thus, a balanced decision between ischemic and bleeding risk should be considered in such cases [33-35].

We do acknowledge that other factors may have been implicated in the patient's ACS, such as his ethnicity and lipoprotein(a) (Lp(a)) levels, which were not measured. While it may appear on the surface as a premature index event due to the absence of an LCx artery, it would be remiss to overlook that South Asian individuals have an inherently elevated risk of CAD, which is not reflected in contemporary risk-estimating equations [36,37]. Additionally, South Asians have the second-highest Lp(a) concentrations amongst ethnic subpopulations, a negative prognosticator for major adverse cardiovascular events (MACE) [38]. Despite no established causal link between ethnicity and CAAs, specific ethnic subpopulations, such as Asians, may display variations in the prevalence of CAAs, such as a malignant course [39]. Our patient did not possess conventional cardiovascular risk factors for his ACS, hence the swift recognition of an alternative etiology, such as CAAs, in this case, an absent LCx artery with an sdRCA.

While routine aspiration thrombectomy is not recommended for all ACS patients, it can be considered in specific situations, such as high thrombus burden, as in our patient. We opted not to aspirate due to operator concerns about precipitating the no-reflow phenomenon (worsening from TIMI 2 with resultant cardiogenic shock, lethal arrhythmias, and lack of advanced hemodynamic support resources) and cerebrovascular events, such as stroke, without a consistent, demonstrable benefit in terms of MACE [40]. Additionally, in a recent meta-analysis, colchicine, when initiated early in addition to guideline-directed medical therapy following ACS, can potentially attenuate medium- and long-term MACE primarily by reducing inflammation, hence our main rationale for its use [41]. He was not instituted on any allergy protocols with oral steroids and antihistamines for his suspected aspirin and NSAID allergies, as his current pharmacotherapies (ticagrelor and apixaban) were considered different classes of agents. There is limited data on aspirin desensitization for patients with coronary artery disease; therefore, it was not immediately considered in the acute setting [42]. Intravascular ultrasound (IVUS) offers a significantly enhanced degree of temporal and spatial discriminative accuracy compared to standard coronary angiography, potentially better delineating the proximal portion of CAAs. OCT is a light-based technology with higher spatial resolution but lower penetration compared with IVUS, and is more useful for defining the coronary luminal surface than for the entire depth of vessel walls and anatomic relationships [43]. Unfortunately, we did not have access to these imaging modalities at our Caribbean location to precisely determine the absence of the LCx artery; therefore, we relied solely on cineangiographic imaging for this determination.

Although this potent antithrombotic combination may be considered off-label in this clinical scenario, as the patient did not have atrial fibrillation, he, after extensive risk-benefit analysis, was in consensus for an initial one-month duration prior to repeat angiography, which eventually revealed complete thrombus dissolution, after which he was continued on ticagrelor monotherapy [43]. This antiplatelet and anticoagulant strategy was highly individualized, given his CAA and suspected aspirin hypersensitivity in the PPCI context. Further large-scale studies are required to elucidate the clinical efficacy and safety of these regimens in high-risk subpopulations, as exhibited in our patient.

## Conclusions

This case report highlights the importance of early identification of CAAs in managing ACS to mitigate against MACE. This patient was treated with CGDMT with an antithrombotic strategy of ticagrelor and apixaban in view of his aspirin hypersensitivity. The successful management of this patient, achieved through tailored therapeutic interventions, underscores the importance of individualized strategies in managing rare CAAs.
